# Radiation‐free or Chemo‐free: Rethinking the Best Strategy for PD‐L1‐High, Unresectable Stage III NSCLC

**DOI:** 10.1002/pro6.70088

**Published:** 2026-09-09

**Authors:** Yongbo Xiang, Nan Bi

**Affiliations:** ^1^ Department of Radiation Oncology, National Cancer Center/National Clinical Research Center for Cancer/Cancer Hospital Chinese Academy of Medical Science and Peking Union Medical College Beijing China

## Abstract

This article discusses the emerging radiotherapy‐free and chemo‐free strategies in PD‐L1–high, unresectable stage III NSCLC, reviewing the Evolution trial and comparing it with existing treatments. We highlight the need for further research to validate these approaches and ensure safe de‐escalation without compromising curability

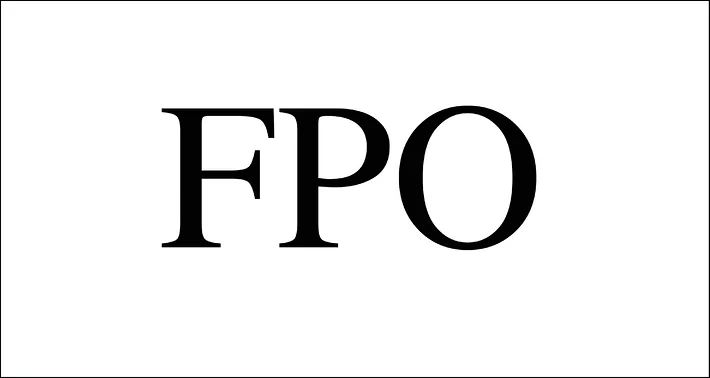

1

The Evolution trial (WJOG11819L)^1^ recently published in *The Lancet Oncology*, provides valuable insights into chemotherapy plus pembrolizumab without thoracic radiotherapy (RT) for patients with locally advanced unresectable NSCLC. The study met its primary endpoint, reporting a 2‐year progression‐free survival rate of 67% and a median overall survival of 44.4 months. The reported overall response rate (ORR) of 81% and complete response (CR) rate of 38% are noteworthy. Although these findings are promising, further validation is warranted to confirm their robustness.

First, the reported efficacy outcomes appear higher than those observed in previous studies. Prior trials evaluating pembrolizumab monotherapy or pembrolizumab combined with chemotherapy reported ORRs ranging from 44.8% to 64.4% and CR rates ranging from 3% to 4.5%.^2^, ^3^, ^4^ By contrast, the Evolution Trial reported an ORR of 81% and a CR rate of 38%, raising questions regarding the consistency and generalizability of these findings. Could the authors clarify whether PET/CT or ctDNA was used in the assessment of treatment response? Including these measures in future evaluations may provide additional insights into treatment efficacy.

Second, the Evolution trial enrolled patients who were considered eligible for definitive chemoradiotherapy but omitted RT. Regarding the timing of local interventions, six patients in this study experienced disease progression, including four with local progression. Of these, three received salvage RT but ultimately died. These findings suggest that salvage therapy after disease progression may not be optimal. Therefore, the optimal timing of local interventions, such as RT or surgery, warrants further investigation to determine the most effective treatment sequence. Importantly, unresectable stage III NSCLC remains a potentially curable disease, for which durable local control is essential. Even in patients with high PD‐L1 expression, the omission of thoracic radiotherapy should be considered cautiously. The integration of immunotherapy with hypofractionated or adaptive radiotherapy may represent a biologically rational compromise that preserves curability, while exploring deescalation.

Phase III studies such as KEYNOTE‐024 and KEYNOTE‐042 demonstrate that high PD‐L1 expression is a strong predictive biomarker of single‐agent immunotherapy efficacy. ^2^, ^3^ This provides a biological rationale for exploring chemotherapy‐free strategies in selected patients with stage III disease. However, concurrent chemoradiotherapy followed by durvalumab remains the evidence‐based standard of care. Therefore, chemotherapy‐free de‐escalation should be considered investigational and ideally evaluated in prospective clinical trials. It is also plausible that extremely high PD‐L1 expression (e.g., ≥90%) may identify a biologically distinct subgroup that is more likely to benefit from treatment de‐escalation. Future trials should consider stratifying patients by PD‐L1 status to refine patient selection. In addition to SPRINT,^5^ which evaluated pembrolizumab combined with risk‐adapted thoracic radiotherapy without chemotherapy in PD‐L1‐high locally advanced NSCLC, other prospective chemo‐free approaches have been explored. For example, the DOLPHIN trial investigated durvalumab plus concurrent curative radiotherapy without chemotherapy,^6^ whereas the SPIRAL‐RT and DUART trials evaluated durvalumab consolidation following radiotherapy alone, particularly in patients who were ineligible for chemotherapy.^7^, ^8^ Collectively, these studies suggest that chemotherapy‐free strategies may be feasible in carefully selected patients; however, they remain early‐phase approaches and should not replace standard chemoradiotherapy‐based treatment.

Grade 3 or higher toxicities reported in this trial, such as neutropenia and leukopenia, are likely chemotherapy‐related, suggesting that some patients may benefit from omitting chemotherapy. As shown in Table 1, the SPRINT trial ^5^(chemotherapy‐free) reported 2‐year efficacy outcomes similar to those of the evolution trial (radiation‐free), with fewer hematologic toxicities and no increase in pneumonitis. Both radiation‐ and chemotherapy‐free strategies are potential options for de‐escalation in stage III NSCLC. When comparing radiation‐free and chemotherapy‐free strategies, evaluation should extend beyond short‐term efficacy and acute toxicity. A radiation‐free approach may avoid late risks such as radiation pneumonitis and cardiotoxicity, whereas a chemotherapy‐free strategy may reduce long‐term chemotherapy‐related complications, including neurotoxicity and renal impairment. These long‐term considerations are particularly relevant for potentially curable patients with prolonged survival. Further head‐to‐head clinical trials are required to validate these strategies.

**TABLE 1:** Comparison of Efficacy and Common Adverse Events between the Evolution and SPRINT Trials

Characteristics	Evolution trial (N=21)	SPRINT trial(N=25)
**Population**	Stage III NSCLC PD‐L1≥50%	Stage III NSCLC PD‐L1≥50%
Median follow‐up time (months)	32.5	22
**PFS**		
Median PFS (months)	Not reached	26.0
1 year PFS (%)	71%^*^	76%
2 year PFS (%)	67%	52%^*^
**OS**		
Median OS (months)	44.4	Not reached
1 year OS (%)	95%^*^	92%
2 year OS (%)	81%	76%
**Common adverse events (Grade≥3)**		
Leukopenia No. (%)	4(19%)	‐
Neutropenia No. (%)	5(24%)	‐
Anemia No. (%)	2 (10%)	1(4%)
Pneumonitis and Pneumonia No. (%)	5(24%)	1(4%)
Grade 5 (deaths) No. (%)	1(5%)	0(0%)

^*^Data not reported in the main text; values were estimated from digitized Kaplan–Meier curves using WebPlotDigitizer (version 5). Cross‐trial comparisons should be interpreted with caution due to differences in study design and patient populations.

NSCLC, Non‐Small‐Cell Lung Cancer; PFS, progression free survival; OS, overall survival

In conclusion, the evolution trial showed promising results for patients with PD‐L1‐high, locally advanced NSCLC, but several important questions remain. These include refining response assessment methods, determining the optimal timing of local treatment, and exploring chemotherapy‐free or radiation‐free strategies. We look forward to future studies that address these questions.

## AUTHOR CONTRIBUTIONS

All authors contributed to the concept and design of this study and approved the version for publication. N. B. and Y. X. drafted the manuscript. N.B. reviewed the manuscript. Each author participated sufficiently in this work to take responsibility for appropriate portions of the content.

## CONFLICT OF INTEREST STATEMENT

The authors declare no conflict of interest.

## FUNDING

This work was supported by National High‐Level Hospital Clinical Research Funding (80102022503).
